# Supplementary figures and images for: Impact of a patient-derived hepatitis C viral RNA genome with a mutated microRNA binding site
Source: PLoS Pathog. 2019 May 10;15(5):e1007467. doi: 10.1371/journal.ppat.1007467 (PMC6530871; doi:10.1371/journal.ppat.1007467)

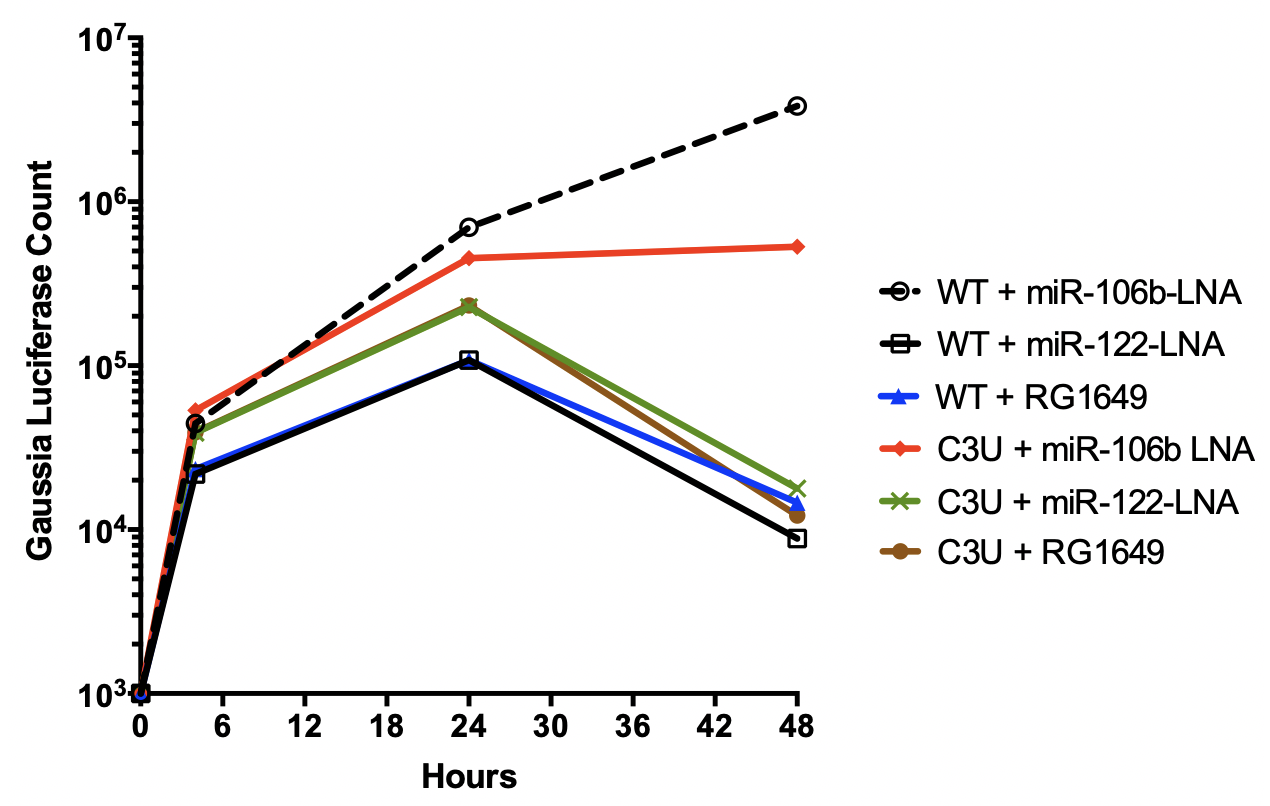

Supplement: S1 Fig — Shown are the non-normalized data from Fig 1B. Viral RNA replication was measured by luciferase production at the indicated time points. (TIF) [file ppat.1007467.s001.tif]

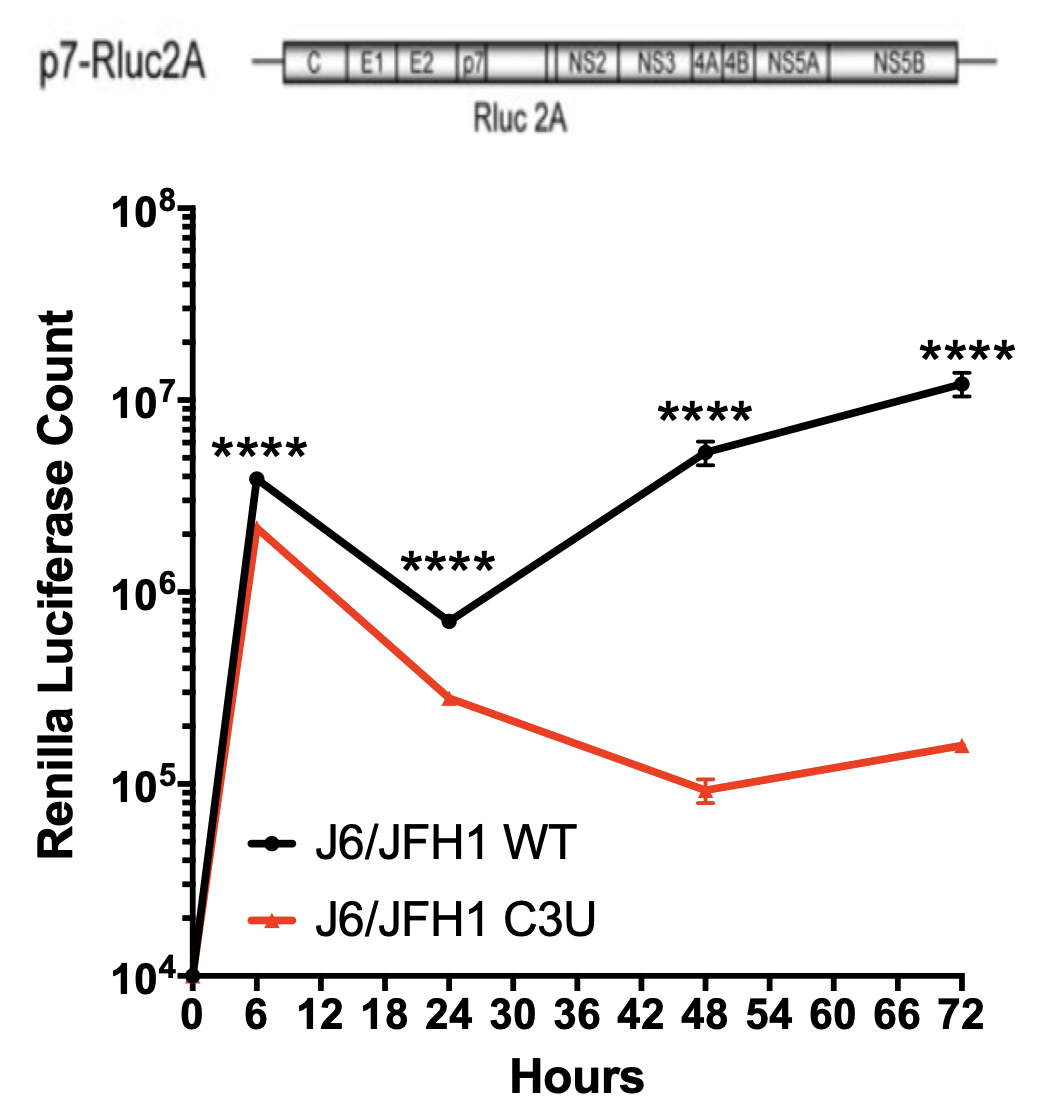

Supplement: S2 Fig — Huh7.5 cells were transfected with wild-type or C3U J6/JFH1 RLuc genomes and RNA replication was determined at the indicated time points. P values are < 0.0001. (TIF) [file ppat.1007467.s002.tif]

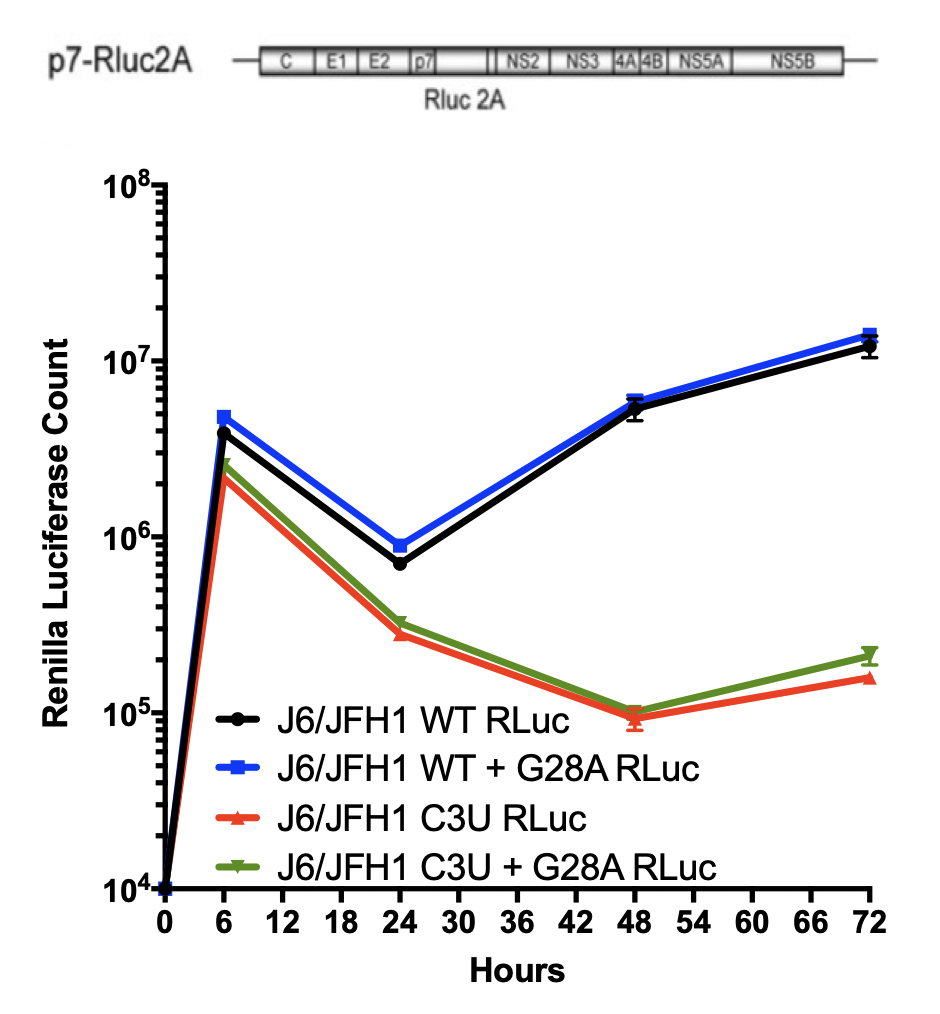

Supplement: S3 Fig — Huh7.5 cells were transfected with chimeric J6/JFH1 RLuc genomes and RNA replication was determined at the indicated time points. P values are < 0.0001. (TIF) [file ppat.1007467.s003.tif]

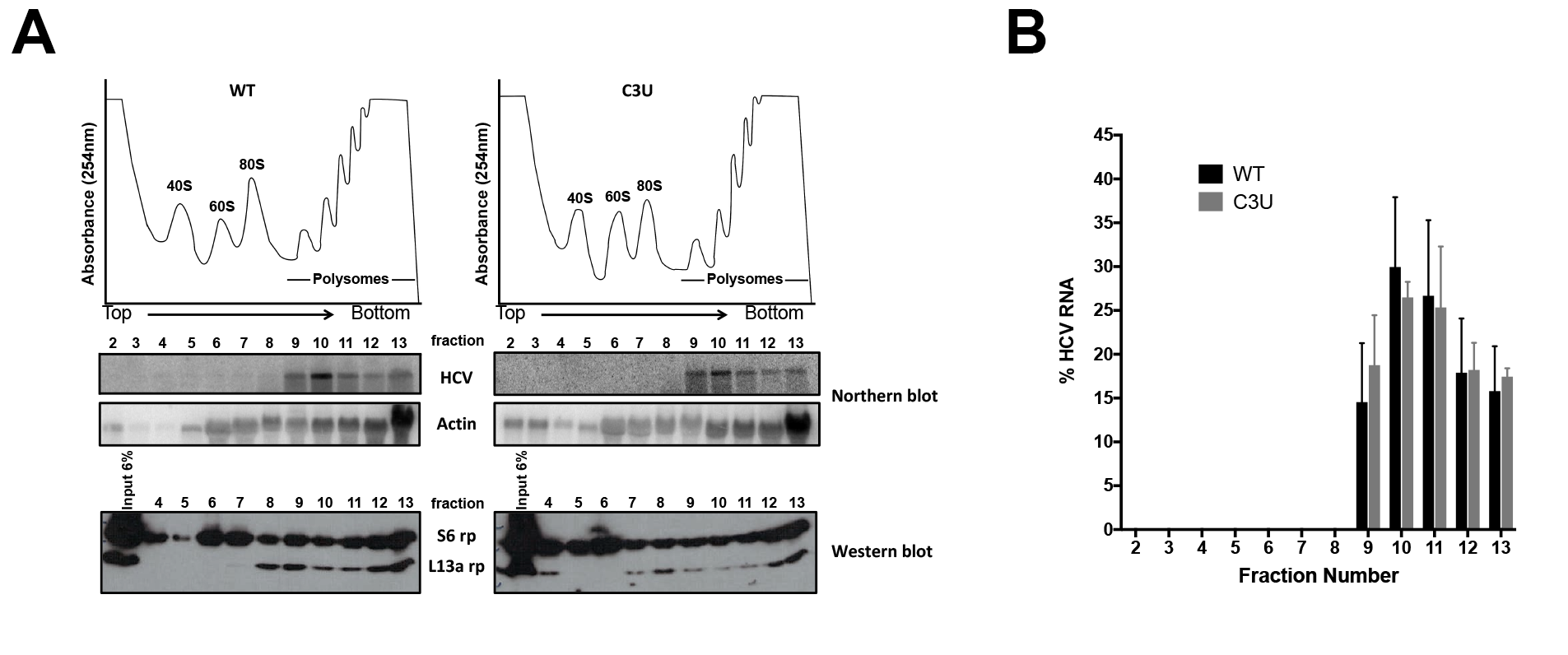

Supplement: S4 Fig — (A) HCV RNA distribution across a sucrose gradient was determined three days following transfection of Huh7.5 cells with wild-type and C3U H77.S3/GLuc RNAs. Polysomal profile trace of lysates separated in 10–60% sucrose gradients. Individual subunits, monosomal, and polysomal peaks are indicated (top). Detection of HCV and actin RNA in sucrose fractions 2 through 13 by Northern blot analysis (middle). Small (S6 rp) and large (L13a rp) ribosomal protein abundances detected by Western blot of total protein isolated from input (6%) and from fractions 4 through 13 (bottom). (B) Percent of HCV RNA distributed across the polysomal gradients of three independent experiments. Error bars display +/- SD. (TIF) [file ppat.1007467.s004.tif]
