# Supplementary material for: Impact of a patient-derived hepatitis C viral RNA genome with a mutated microRNA binding site
Source: PLoS Pathog. 2019 May 10;15(5):e1007467. doi: 10.1371/journal.ppat.1007467 (PMC6530871; doi:10.1371/journal.ppat.1007467)
Supplement: S1 Methods — (DOCX) [file ppat.1007467.s006.docx]

**Supporting Information Supplemental methods**

**Polysomal profiling**

Huh7.5 cells were plated at a density of 5x10^6^ one day before HCV IVT RNA transfection. The following day, cells were transfected with 10 μg of HCV GLuc wild-type or C3U RNAs. Three days post transfection, 100 μg/l of cycloheximide was added to each plate. Cells were incubated for three minutes at 37°C, followed by wash with ice-cold PBS, and lysis directly on plate by adding 600 μl of polysomal lysis buffer (20mM Tris pH 7.5, 150mM NaCl, 5mM MgCl_2_, 1mM DTT, and 100 μg/mL of cycloheximide). Lysates were incubated on ice for ten minutes with periodic agitation and clarified after sedimentation at 14,000 rpm for 10 minutes at 4°C. RNA abundances in the supernatants were quantified by Nano-drop and 250 μg of cleared lysates were layered onto 10-60% sucrose gradients. Gradients were centrifuged at 35,000 rpm at 4°C in an SW41 rotor for 165 minutes and samples were fractionated with an Isco Retriever II/UA-6 detector system. RNA was extracted by adding an equal volume of acid phenol/chloroform to each fraction, centrifuged at 14,000 rpm for 15 minutes at 4°C. To remove any possible contaminants, an equal volume of chloroform was added to the aqueous fraction and centrifuged at 14,000 rpm for 10 minutes at 4°C. RNA was precipitated overnight by addition of 1 volume of isopropanol to the aqueous fraction and stored at 20°C. RNA was pelleted at 14,000 rpm for 15 minutes at 4°C, washed twice with 75% ethanol, dried for 5 minutes at room temperature, and dissolved in water. HCV and actin RNA abundances across all fractions were determined by Northern blot analysis. HCV RNA abundances per fraction were determined from three independent experiments and quantified using ImageJ.

**Mass spectrometry analyses**

Mass spectrometry analyses were performed on duplicate samples (WTA, WTB, C3UA, C3UB) and on viral RNA lacking the BoxB motif. SAINT analysis was used to detect the distinct binding of proteins to WT and C3U RNAs [33]. Correlation analysis results (Spearman) showed a very high degree of correlation between samples (WT & C3U):

C3UA C3UB WTA WTB

C3UA 1.0000000 0.9587977 0.9665938 0.9529387

C3UB 0.9587977 1.0000000 0.9881529 0.9914419

WTA 0.9665938 0.9881529 1.0000000 0.9873310

WTB 0.9529387 0.9914419 0.9873310 1.0000000

See Supplemental Information (Microsoft Excel File) for the complete data set .
